# Best practice guidelines for clinical and radiological assessment of patients with femoroacetabular impingement. Results from the ISHA International Delphi Consensus Project—Phase 2

**DOI:** 10.1093/jhps/hnad028

**Published:** 2023-11-23

**Authors:** Sarkhell Radha, Jonathan Hutt, Ajay Lall, Benjamin Domb, T Sean Lynch, Damian Griffin, Richard E Field, Josip Chuck-Cakic

**Affiliations:** Trauma and Orthopaedics, Croydon University Hospital, 530 London Road, London CR7 7YE, UK; Al-Kindy University, Mohamed Al-Qasim Expy, Baghdad, Iraq; Trauma and Orthopaedics, University College London Hospital, 235 Euston Road, London NW1 2BU, UK; America Hip Institute, 999 E Touhy Ave # 450, Chicago 60018, USA; America Hip Institute, 999 E Touhy Ave # 450, Chicago 60018, USA; Northwestern University, 633 Clark Street, Chicago, IL 60208, USA; University Hospitals Coventry and Warwickshire, Clifford Bridge Road, Coventry CV2 2DX, UK; South West London Elective Orthopaedic Centre, Dorking Road, London KT18 7EG, UK; Rosebank Centre for Sports Medicine and Orthopaedics and Fourways Life Hospital, 9 Sturdee Avenue, Johannesburg, South Africa

## Abstract

In 2018, the International Society for Hip Preservation Surgery (ISHA) initiated a series of Delphi consensus studies to identify the global hip preservation community’s current opinion on best practices for different facets of hip preservation surgery. Arthroscopic procedures to treat hip pathologies, such as femoroacetabular impingement syndrome (FAIS) are now established in mainstream orthopaedic practice. This study establishes recommendations for the investigation of patients with suspected FAIS. The investigation has focused on the three phases of the diagnostic process—patient history, physical examination and special investigations. Our expert panel consisted of 174 international orthopaedic surgeons with expertise in hip preservation surgery, thereby making recommendations generalisable across the globe. After three rounds of survey and analysis with 174 participants per round, our study achieved consensus at a minimum agreement threshold of 80.0% on 55 statements pertaining to the assessment of patients with FAIS. We encourage our junior and senior hip arthroscopy colleagues internationally to consider these statements both to standardize the clinical and radiological assessment of patients with FAIS and to aid in the design of future research.

## INTRODUCTION

In 2018, the International Society for Hip Preservation Surgery (ISHA) initiated a series of Delphi consensus studies to identify the global hip preservation community’s current opinion on best practices for different facets of hip preservation surgery. The first ISHA study was inspired by the work presented by Dr Sean Lynch at the 2018 Vail Hip Arthroscopy meeting. The investigation provided the first USA consensus-based Best Practice Guidelines from 15 high volume USA hip arthroscopists and was published in July 2019 [1].

Recognising the value of Lynch’s work, the 2017–2018 President of ISHA, Dr Josip Chuck Cakic, proposed that a global ISHA consensus study would fulfil the WHO recommendation that standardized interventions should be developed for patient safety utilizing evidence-based processes and best practice initiatives [2]. The study aim was to utilise a Delphi consensus method to identify whether global consensus-based guidelines for arthroscopic intervention for femoroacetabular impingement syndrome (FAIS) could be developed. The Delphi process is a structured communication technique that uses a systematic, interactive forecasting method through a panel of experts. The experts assess statements in multiple rounds of the study process. After each round, research facilitators use an anonymised summary of the responses to modify statements where there is disagreement for the next iteration—these statements are then presented back to the panel. During this process, the range of the responses diminishes and the group converges towards a consensus on each topic.

The first ISHA study focused on the surgical management of FAIS and was published in December 2019 [3]. We now report the results of the second phase of the ISHA initiative. This study establishes recommendations for the investigation of patients with suspected FAIS. The purpose of this study was to establish recommendations for the investigation of patients with suspected FAIS, focusing on the three phases of the diagnostic process—patient history, physical examination and radiological assessment.

## SUBJECTS AND METHODS

### Study participants

All members of ISHA that are listed as surgeons with a special interest in hip preservation surgery were invited by email to take part in the study via a link to an online questionnaire. Those who agreed to participate provided details of their geographical region of practice, total number of years in practice and annual, as well as career total, number of hip arthroscopies performed.

### Study design

This study focussed on the clinical and radiographic assessment of patients with FAIS. The same expert study group of ISHA members from the first ISHA Delphi Consensus Study collated a list of potential topics to include along with indicated initial statements. These were reviewed by the ISHA Board via an online survey tool. The board members were asked to rank the topics with regard to their relevance for inclusion on a scale of 1 to 10, and then to say whether they would put the statement forward in its current form or to offer further modifications or suggestions.

These initial statements were put to the participating ISHA members in the weeks preceding the 11th Annual Scientific Meeting in Madrid, 2019. Online questionnaires were conducted using the Mesydel platform (Seraing, Belgium). Individual encrypted login details were used and participants were asked to consider each statement using a four-point Likert scale of strongly disagree, disagree, agree and strongly agree. If respondents disagreed, they were invited to comment and offer an alternative statement to which they could agree. The responses were then analysed anonymously. The consensus agreement level was set at 80.0%. The content validity ratio (CVR) as described by Lawshe in 1975 was not used in this project. CVR is a linear transformation of a proportional level of agreement. The main benefit of CVR is to readily indicate whether the level of agreement among panel members exceeds 50%. However, as agreement was agreed at 80% or higher, CVR was not needed.

**Fig. 1.**
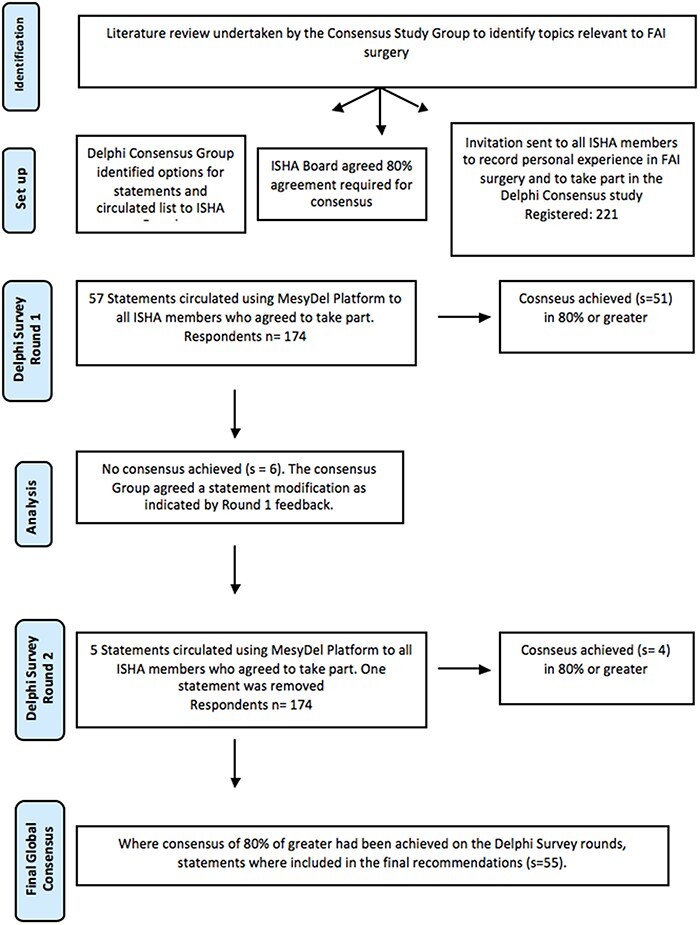
The overall study design is summarised in Fig. 1.

After each round, statements that had achieved 80% consensus at either half of the Likert scale were recorded and removed from subsequent survey rounds. The responses to those that did not reach consensus were reviewed by the study group and used as a basis to reword the statements, which were then put forward for a further questionnaire round. For all subsequent rounds, the voting results of the previous rounds were visible to the participants. The study design is outlined in Fig. 1.

## RESULTS

A total of 221 ISHA members from seven global regions (Table I), with a mean of 12.1 years (range 0–37 years) in practice registered to participate.

**Table I.** Number of ISHA respondents

Continents	Number of ISHA respondents
Australia and New Zealand	21
Africa	5
North America	90
South America	19
Asia	18
Europe and Middle East	63
Other	5

The mean number of hip arthroscopies performed annually was 118 (range 2–450), with a total number of 21 408 per year and 189, 254 overall.

Two rounds of the Delphi process were conducted, with a complete response rate from 174 of the 221 registered participants (78.7%) in each round.

Of the initial set of 57 statements, 51 reached consensus in the first round. Following a review of the results and additional comments, the six statements that did not reach consensus were re-circulated to the ISHA Delphi Consensus Group as shown in Table II. Body mass index was moved from history into clinical examination when re-circulated. ‘Turned lateral views’ in the radiographic investigation section was removed due to different regions using different terms for this view. Following the second round, only one statement did not reach consensus: The following examination tests are useful in the evaluation of a patient with FAIS—ligamentum teres (LT) stress test (J O’Donnell, 2014) [4]. The complete set of consensus statements is shown in Table III.

**Table II.** Statements which did not receive consensus of 80% or more and modified statements

Statements which did not receive consensus	Consensus	Modified statements	Consensus
1. History		1. History	
Gender	76.7	Gender	90.1
Body mass index	77.6	Medication for other conditions	87.7
Medication for other conditions	79.9	Venous thromboembolism assessment and treatment	79.2
Venous thromboembolism assessment and treatment	76.4		
		2. Clinical examination	
2. Clinical examination		Body mass index	89.2
Ligamentum teres stress test (J O’Donnell, 2014)	63.9	Ligamentum teres stress test (J O’Donnell, 2014)	43.1
3. Investigations		3. Investigations	
Turned lateral views	64.6	Turned lateral views	Removed

**Table III.** The complete set of consensus statements

Statement	Consensus
History	
Age	95.4
Gender	90.1
Level of current activities and expectations	98.3
Pain characteristics—distribution	99.4
Pain characteristics—precipitating/relieving factors	99.4
Pain characteristics—activity related/positional	98.3
Duration of symptoms	97.1
Associated symptoms—snapping/popping	81.6
Associated symptoms—giving way/instability/catching	90.2
Impact on function and quality of life	99.42
Analgesia for hip pain	90.7
Medication for other conditions	87.7
Prior childhood hip disorders	97.7
Venous thromboembolism assessment and treatment	79.2
Family history of hip conditions	86.8
Previous physiotherapy for FAIS	93.1
Previous injections to the hip joint	94.8
Previous hip surgery	98.3
Previous history of trauma	91.3
Clinical examination	
Body mass index	89.2
Gait	91.8
Range of motion and fixed deformity	100
Generalised hypermobility (Beighton score)	97.6
Anterior apprehension test	87.7
Impingement signs (FADIR/FABER)	100
Labral stress test (e.g. hip distraction test)	82.4
Investigations	
Antero-posterior pelvis radiograph	100
Cross table true lateral in internal rotation radiograph or Dunn view	91.0
False profile radiograph if CT unavailable	91.0
Measurements/assessment of radiographs	
Evidence of joint degeneration (Tonnis or Kellagran and Lawrence)	100
Lateral centre edge angle	100
Anterior centre edge angle	98.2
Sourcil angle	99.4
Head-neck asphericity	100
Femoral neck/shaft angle	95.8
Labral calcification	96.9
Profunda/protrusio	98.2
Crossover sign	95.2
Posterior wall sign	96.4
Subchondral cysts	100
Specialised radiological investigations: CT scan if available and indicated CT arthrogram	
When available, a CT scan is a useful for evaluation of a patient with FAIS?	85.03
The following features are useful to evaluate from a CT—acetabular rim cyst	95.1
The following features are useful to evaluate from a CT—acetabular version	95.8
The following features are useful to evaluate from a CT—femoral version	99.4
The following features are useful to evaluate from a CT—acetabular coverage	96.3
The following features are useful to evaluate from a CT—femoral cam morphology	97.8
The following features are useful to evaluate from a CT—acetabular pincer morphology	96.4
The following features are useful to evaluate from a CT—anterior inferior iliac spine avulsion (AIIS)	96.4
The following features are useful to evaluate from a CT—subchondral cysts	95.2
Specialised radiological investigation: MRI scan or MR arthrogram if available or indicated	
When available, an MRI scan is a useful imaging study for evaluation of a patient with FAIS?	95.8
The following features may be useful to evaluate from an MRI—labral pathology	99.4
The following features may be useful to evaluate from an MRI—avascular necrosis (AVN)	99.4
The following features may be useful to evaluate from an MRI—chondral surfaces	98.2
When available, do you think the information from 3D motion analysis is valuable in patients with FAIS?	82.3
Does performing diagnostic injections in selected patients with FAIS add value to your clinical practice?	95.7

## DISCUSSION

We present the second phase of the ISHA international consensus project—clinical and radiological assessment of patients with FAIS. Our expert panel consisted of 174 international orthopaedic surgeons with expertise in hip preservation surgery, thereby making recommendations generalisable across the globe. The Delphi technique allowed all the participants to respond individually and anonymously, thus avoiding many problems associated with live workshops or focus groups, where strong characters or the ‘majority view’ can overwhelmingly dominate. Our chosen method also offered participants the opportunity to review and revise their opinions throughout the process, leaving time for thoughtful reflection and consideration. Three main aspects of assessment were considered in this study: history, clinical examination and investigations. After three rounds of survey and analysis with 174 participants per round, our study achieved consensus at a minimum agreement threshold of 80% on 55 statements pertaining to the assessment of patients with FAIS.

The online Delphi consensus technique appears to be a well-established method of harnessing opinions among a diverse group of experts regarding practice-related problems. Our Phase I study highlighted this technique’s use in orthopaedic surgery, within the sports medicine and hip preservation literature [5, 6]. More recently in 2019, the ISHA Delphi study group published the first international best practice guidelines in ‘Journal of Hip Preservation Surgery’ entitled ‘Best Practice Guidelines for Arthroscopic Intervention in Femoroacetabular Impingement syndrome’. This second study has 174 participants providing their expertise and, to the author’s knowledge, is the largest Delphi consensus study conducted regarding the clinical evaluation of patients with FAIS [1].

Like the previous study, inherent limitations of the Delphi technique are acknowledged by authors of this study. Potential attrition between rounds was mitigated by participants knowing this was a multi-phase study and all were encouraged to continue participation until completion. However, with anonymity in a study such as this, an understood limitation includes potential absence of stimulation and cross-fertilization of ideas that can occur when people meet face-to-face or in a group setting such as a conference. Lastly, variability in participants’ expertise in arthroscopic hip surgery can be seen as a limitation, or instead, as a study strength since consensus among varying surgical skill levels is imperative when recommending and implementing consensus statements to an international patient population within a relatively new field of surgical practice.

Preoperative assessment typically consists of a thorough history, physical exam and diagnostics such as imaging in the case of FAIS [7]. The authors would like to highlight certain areas covered by the consensus statements in relation to history, clinical examination and radiological assessment to the significant debate within the current literature.

## HISTORY

History taking is an important aspect in successful physician–patient interaction. Gathering important information from the patient’s medical history is necessary for effective clinical decision in FAIS [8]. Table III shows all statements that achieved consensus at above 80% threshold for this survey. As expected, many statements are consistent with thorough history taking in any new patient presenting for evaluation; level of activity, pain, symptom duration, associated findings, impact on activities of daily living, analgesic medications, prior hip conditions or systemic ailments, family history, history of non-surgical or surgical treatment. Some statements studied extensively in the literature are further expanded on below.

### Age—95.4%

This finding was consistent with Mittag et al (2015) who reported knowing a patient’s age has a relevant impact on various radiographic parameters to detect FAIS and hip dysplasia [9].

While femoral caput collum-diaphyseal (CCD) angle decreases only marginally, acetabular coverage increases considerably over time [10]. The age of onset to perform a hip arthroscopy has been a matter of debate in the orthopaedic literature. Most studies have reported only short-term outcomes for patients above the age of 50 [11–15]. Although improvements in patient reported outcomes and overall patients’ satisfaction were reported favourably in patients of over 50 years old, conversion rate to THA varied from 16% to 52%. In 2018, a conversion rate of 27.7% to THR was reported at a minimum of 5-year follow-up following hip arthroscopy. The author of this study recommended appropriate patient selection to include age, when assessing patients for possible arthroscopic hip surgery [16].

### Gender—90.1%

Gender differences have shown to affect the pathoanatomy of patients with FAIS. Younger males are often seen to have decreased femoral head–neck offset, a non-spherical femoral head, or a decrease in the angle of the head and neck of the femur relative to the femoral condyles (femoral retrotorsion) [16–20]. These morphologies have been seen to result in symptomatic CAM type FAIS at a higher rate compared with female gender. On the other hand, Pincer-type FAIS has been reported to be more prevalent in women [21]. Although gender did not receive <80% consensus in the first round, it subsequently went on to receive consensus of 90.08% in the second round of this study.

### VTE—80.2%

Orthopaedic surgical procedures may carry increased risk for the development of venous thromboembolism (VTE) due to temporary endothelial dysfunction, venous stasis as a result of patient immobilization during the recovery period, and a possible hypercoagulable state, which is patient dependent. Insufficient evidence exists to support whether anti-VTE chemoprophylaxis should be administered to patients undergoing primary hip arthroscopy for FAIS. Due to the life-threatening character of this complication, it is fundamental to be aware of the patients VTE status. Although VTE assessment and treatment did not receive <80% consensus in the first round, it subsequently went on to receive consensus of 80.2% in the second round of this study [22]. The ISHA Delphi consensus group recommends VTE assessment to be part of history taking in patients with FAIS considered for surgical intervention.

### Pain—80.0%

In 2003, Ganz et al described pain to be the primary symptom of FAIS. The detailed description of pain with its relationship to activities of daily living has been reported to be a crucial aspect of history taking in patients with FAIS. In most patients who seek treatment for FAIS, symptoms are not mild or subtle. They are often severe and limiting in everyday life. The panel felt that this is especially important because patients are usually young, active adults involved in the workforce. Symptoms of FAIS can therefore lead to a significant cost burden for society, in addition to being individually debilitating. Consensus of 80% or more was achieved in all pain and quality of life related statements in this Delphi consensus study [23–26].

### Previous injections to the hip joint—94.8%

Image-guided injection of local anaesthetic, with or without corticosteroids, can help to identify whether pain generators are from intra- or extra-articular regions of the hip joint. Previous studies, including the Warwick Agreement have reported the usefulness of knowing if a patient has had an image-guided injection (X-ray or ultrasound) in the past. Consensus of >90% was achieved in favour of image guided injection in FAIS during this Delphi consensus study [4, 27–29].

### Clinical examination

All clinicians are taught that a proper examination of the patient is critical to understanding the root cause of ailment. This teaching holds true for patients presenting with FAIS of the hip.

### Gait—91.8%

Few studies have correlated radiographic FAIS morphology with gait abnormalities. Farkas et al in 2015 undertook a study looking at preoperative gait evaluation within 1 month of the patients’ scheduled surgical date. Results from this study found that variability in gait pattern was accounted for by a large amount of variation in alpha angles among patients studied. In addition, other studies have shown that gait and lower extremity kinematic parameters correlate with radiographic FAIS morphology in symptomatic patients [30], such as intoeing gait in patients with excessive femoral anteversion. In this current study, gait analysis achieved 91.77% consensus for patients presenting with FAIS.

### Hypermobility—97.6%

Patients with hypermobility syndrome tend to have increased hip range of motion, and therefore are at an increased risk of placing their hip in positions of potential impingement, even when there are no osseous abnormalities. Furthermore, hypermobility has been shown to increase the risk of intrarticular chondrolabral injury in dancers and gymnasts, due to increased range of motion and potential subluxation [31–33]. These injuries might predispose patients to a combination of instability and impingement. Novel arthroscopic surgical strategies in hypermobile patients with FAIS include enhanced capsular management techniques, such as repair or plication, in an effort to minimize joint translation and potential for re-injury [34–36]. In this study, 97.64% of participants agreed that hypermobility testing should be done during physical examination of patients presenting with FAIS.

### Anterior apprehension test—87.7%, impingement sign—100%, labral stress test—82.4%

A succinct, systematic approach to clinical examination is warranted while assessing patients with FAIS [37]. This is particularly the case for diagnosing intra‐articular pathology of the hip such as labral pathology, which, unfortunately continues to suffer from the lack of high quality evidence in support of various examination and testing measures. Consensus of 80% or more was achieved for statements involving anterior apprehension test, impingement signs (FADIR/FABER) and labral stress test (e.g. hip distraction test) when assessing the patient with FAIS. These clinical tests when used together may be more comprehensive and reliable in helping to diagnose intra-articular hip pathologies [38].

### Ligamentum teres test—63.9%

The LT has attracted greater interest over recent years due to enhancing diagnostics and surgical expertise of hip arthroscopy [39]. A LT injury is a common finding at the time of arthroscopic hip surgery in patients with chronic groin and hip pain; however, LT tears have been difficult to identify before surgery. There have been no unique features identified on history assessment, physical examination, or imaging that reliably identifies injury of the LT preoperatively. The LT test is an effective way of assessing the presence of LT tears with moderate to high inter-observer reliability. In addition to an LT tear, the presence of a pincer lesion, or labral tear requiring repair, have been shown to be associated with a positive LT test result [40]. However, after two rounds of Delphi analysis, the LT stress test did not achieve <80% consensus for use in evaluating a patient with FAIS. Therefore, it has not been included in the final best practice guidelines for clinical and radiological assessment of patients with FAIS. Reflecting what is published in the current literature, our study participants reached consensus that there is currently no agreement on the LT test (J O’Donnell, 2014).

## INVESTIGATIONS

### Radiographic investigations—> 80%

Multiple imaging modalities, including radiographs, computed tomography (CT) and MRI or magnetic resonance arthrogram (MRA), are used in the preoperative evaluation of FAIS [40]. Plain radiographs have often been used as an initial imaging modality to assess for pincer or cam lesions, hip dysplasia, or advanced osteoarthritis [41]. Antero-posterior, false profile and cross table true lateral in internal rotation radiographs, or the Dunn lateral view, are widely used as an initial screening tool where several measurements are generally performed. Technical variability due to technicians or imaging quality can limit the accuracy and reproducibility of radiographic measurements worldwide; however, starting with plain film imaging has reached consensus of >90%, as seen in Table III [42]. CT and MRI scan have been shown to provide a more accurate alternative to assess femoral and acetabular morphology, with or without 3D reconstructions [43]. MRI or MRA scan is superior to other modalities when evaluating soft tissue injuries in patients with FAIS. It allows direct evaluation of articular cartilage and labral pathology, and can assist in optimizing indications as well as patients’ selection for different surgical strategies [44]. Advanced diagnostic imaging has been recommended as a necessary component of a comprehensive approach to the diagnosis of FAIS, achieving >80% consensus in both CT and MRI evaluations [45].

### Dynamic 3D motion analysis—82.3%

Gait analysis, as aforementioned, has utility in evaluating patients with FAIS. However, femoral head asphericity, and pelvic tilt abnormalities can influence gait function, making assessments less accurate. Dynamic 3D motion analysis, where available, has recently helped mitigate some inaccuracies by evaluating the patient in three planes of motion, using computer technology to analyse the data objectively [46].

## CONCLUSION

This Delphi consensus study involved experienced arthroscopic hip surgeons from a diverse international hip preservation community. It is interesting the consensus from this study is in line with the known literature and there is nothing inconsistent or unknown. It is useful to know and appreciate that orthopaedic surgeons are following the known literature. Three main areas in the arthroscopic treatment of FAIS were considered: history, examination and investigations. After two rounds, 55 consensus statements were produced. We encourage our junior and senior hip arthroscopy colleagues internationally to consider these statements both to standardize the clinical and radiological assessment of patients with FAIS, and to aid in the design of future research. In conclusions, it would be beneficial to have a software tool and a global registry that allows clinicians to record the clinical data recommended by the consensus both to facilitate good clinical process and to facilitate pooled outcome analysis.

## DATA AVAIILABILITY

The data that support the findings of this study are available from the corresponding author, [S.R.], upon reasonable request.
